# *Staphylococcus aureus-*Cure-Associated Antigens Elicit Type 3 Immune Memory T Cells

**DOI:** 10.3390/antibiotics11121831

**Published:** 2022-12-16

**Authors:** Kamila R. Santos, Fernando N. Souza, Eduardo M. Ramos-Sanchez, Camila F. Batista, Luiza C. Reis, Wesley L. Fotoran, Marcos B. Heinemann, Adriano F. Cunha, Mussya C. Rocha, Angélica R. Faria, Hélida M. Andrade, Mônica M. O. P. Cerqueira, Magnus Gidlund, Hiro Goto, Alice Maria M. P. Della Libera

**Affiliations:** 1 Veterinary Clinical Immunology Research Group, Departamento de Clínica Médica, Faculdade de Medicina Veterinária e Zootecnia, Universidade de São Paulo, São Paulo 05508-270, Brazil; 2 Programa de Pós-Graduação em Ciência Animal, Universidade Federal da Paraíba, Areia 58397-000, Brazil; 3 Laboratório de Soroloepidemiologia e Imunobiologia, Instituto de Medicina Tropical, Universidade de São Paulo, São Paulo 05403-000, Brazil; 4 Departamento de Salud Publica, Facultad de Ciencias de La Salud, Universidad Nacional Torino Rodriguez de Mendonza de Amazonas, Chachapoyas 01001, Peru; 5 Laboratório de Genética, Instituto Butantã, Universidade de São Paulo, São Paulo 05503-900, Brazil; 6 Departamento de Medicina Veterinária Preventiva e Saúde Animal, Faculdade de Medicina Veterinária e Zootecnia, Universidade de São Paulo, São Paulo 05508-270, Brazil; 7 Departamento de Tecnologia e Inspeção de Produtos de Origem Animal, Escola de Veterinária, Universidade Federal de Minas Gerais, Belo Horizonte 31270-010, Brazil; 8 Departamento de Parasitologia, Instituto de Ciências Biológicas, Universidade Federal de Minas Gerais, Belo Horizonte 31270-901, Brazil; 9 Laboratório de Parasitologia Clínica, Faculdade de Ciências Farmacêuticas, Universidade Federal de Alfenas, Alfenas 37130-000, Brazil; 10 Departamento de Imunologia, Instituto de Ciências Biomédicas, Universidade de São Paulo, São Paulo 05508-900, Brazil; 11 Departamento de Medicina Preventiva, Faculdade de Medicina, Universidade de São Paulo, São Paulo 01246-903, Brazil

**Keywords:** vaccine, T cell response, IL-17A, intramammary infection, *Staphylococcus aureus*, mastitis, recombinant antigens, dairy cow

## Abstract

Background: *Staphylococcus aureus* is one of the most frequently major mastitis pathogens that cause clinical and subclinical mastitis worldwide. Current antimicrobial treatments are usually ineffective, and the commercially available vaccines lack proven effectiveness. The immunological response elicited by the recombinant *S. aureus*-cure-associated proteins phosphoglycerate kinase (PGK), enolase (ENO), and elongation factor-G (EF-G) in combination with the granulocyte-macrophage colony-stimulating factor (GM-CSF) DNA vaccination was studied in this work. Methods: Here, twenty-three C57BL/6 mice were divided into four groups and vaccinated with: G1: none (control); G2: GM-CSF DNA plasmid DNA vaccine; G3: the combination of EF-G+ENO+PGK; and G4: the combinations of EF-G+ENO+PGK proteins plus GM-CSF plasmid DNA vaccine. After 44 days, spleen cells were collected for immunophenotyping and lymphocyte proliferation evaluation by flow cytometry upon *S. aureus* stimulus. Results: Immunization with the three *S. aureus* recombinant proteins alone resulted in a higher percentage of IL-17A^+^ cells among CD8^+^ T central memory cells, as well as the highest intensity of IL-17A production by overall lymphocytes indicating that the contribution of the combined lymphocyte populations is crucial to sustaining a type 3 cell immunity environment. Conclusion: The immunization with three *S. aureus-*cure-associated recombinant proteins triggered type 3 immunity, which is a highly interesting path to pursue an effective bovine *S. aureus* mastitis vaccine.

## 1. Introduction

Bovine mastitis is the costliest disease in any dairy farm, causing a pronounced reduction in milk production and quality. Furthermore, mastitis implies approximately 70% of the antimicrobial usage in dairy farms, raising serious public health concerns [1]. *Staphylococcus aureus* is one of the most frequently associated bacteria that cause clinical and subclinical mastitis worldwide [2,3]. Because of its pathogenicity, contagiousness, likely persistence of intramammary infections (IMIs), and refractoriness to the current antimicrobial therapy, this mastitis pathogen remains a major challenge for the dairy industry worldwide [3]. Besides that, *S. aureus* is a multi-host bacterial pathogen and may infect a distinct host-species population, constituting a major menace to public health and food security [4,5]. In this context, *S. aureus* mastitis points to an urgent need for innovation and searches for effective alternative approaches to its treatment and prevention. 

Thus, it would be valuable to have an alternative approach that increases the likelihood of the cure of an existing infection in an integrated approach with the antimicrobial treatment [6] or of the prevention of new *S. aureus* infections. We would emphasize that considering the contagious behavior of this pathogen [2], any alternative measure that increases the quarter cure of an IMI may result in lower exposure, decreasing the likelihood of a healthy quarter becoming infected, and consequently reducing the transmission rates and the prevalence of this pathogen in the herd [1]. 

In a previous study, we searched *S. aureus*-derived proteins [7] using sera from *S. aureus* IMI-cured animals, aiming selection of proteins likely related to cure. With this immunoproteomic approach, we revealed three staphylococcal proteins: enolase (ENO), phosphoglycerate kinase (PGK) and elongation factor-G (EF-G). In this latter study, we assumed that antibodies from chronically infected dairy cows clinically and bacteriologically cured *S. aureus* IMIs, after immunization with a commercial inactivated vaccine, recognized *S. aureus* proteins used in the present study that may translate into a therapeutic and/or preventive vaccine of relevance. The first *S. aureus* protein, the so-called ENO, is a conserved moonlighting protein that is expressed in situations when a critical nutrient (e.g., iron) becomes extremely scarce [8], which is the case in the mammary gland environment, especially during mastitis [9]. This protein is essential for bacterial growth and plays an important role in several steps of the infection cycle, with a likely major role in glycolysis, a critical pathway for bacterial energy supply [10,11]. Furthermore, ENO mediates the binding of *S. aureus* to laminin and collagen I guiding the mechanism of invasion and dissemination. Initially, it enables *S. aureus* to bind to the extracellular matrix, initiating tissue colonization, followed by plasminogen activation and laminin degradation, which cause the extracellular matrix destruction, favoring bacterial invasion and spread [8,12,13]. Further, ENO is involved in cell wall biosynthesis and resistance to antimicrobials and had a critical role in biofilm formation [8,11,14]. Similarly, PGK is also a moonlighting protein that plays an important role in *S. aureus* energy metabolism. Its inhibition negatively impacts the bacterial population during biofilm formation. In addition, this staphylococcal protein may be involved in antimicrobial resistance, acts as an adhesion protein [15] and is a potent neutrophil activator with the ability to trigger degranulation. Besides, it dampens the classical and alternative complement pathways [16]. Lastly, the EF-G belongs to the GTPase superfamily that catalyzes two different steps of protein synthesis, and it is used as an energy source, for protein synthesis and cell growth [17,18,19,20]. Even though the molecular functions of these proteins are not precisely understood, it is reasonable to hypothesize that the induction of the host immune response by these proteins may represent an exciting and promising approach for vaccine development. The animals that have successfully eliminated the chronic IMIs by *S. aureus* produce specific antibodies against the abovementioned *S. aureus* proteins showing induction of functioning adaptive humoral responses. This observation encouraged us to study their effect on T-cell-mediated immunity which is considered more and more crucial for solid protection against *S. aureus* infection [21]. Furthermore, some studies have encouraged the use of a granulocyte-macrophage colony-stimulating factor (GM-CSF) DNA vaccine as a powerful immunoadjuvant [22,23] that could be related to T-cell-derived IL-17 production [24,25].

It should be highlighted that there is growing evidence that T cell immunity, especially T-cell-derived interleukin (IL)-17, plays a pivotal role in protection against *S. aureus* infections [24,26,27]; therefore, a fine-tuned characterization of T cell immunity elicited by the *S. aureus* proteins above is proposed here. Thus, in the present study, we investigate the immune response induced by three recombinant proteins of *S. aureus*, EF-G, ENO and PGK in combination with GM-CSF DNA vaccine in mice for future development of use in the cow.

## 2. Materials and Methods

### 2.1. Production of S. aureus Recombinant Proteins

The amino acid sequences encoding to the EF-G (Genbank gi|395759321), PGK (Genbank gi|446997488), and ENO; (Genbank gi|447044500) *S. aureus* antigens were codon-optimized for *Escherichia coli* expression and synthesized by Genscript (Piscataway, NJ, USA). The synthetic genes were cloned into the pUC57 vector and subsequently were sub-cloned into the pET28a expression vector [24]. Recombinant plasmids were used to transform the expression strain *E. coli* BL21-Star™ (DE3) as previously described [24]. Concisely, kanamycin plates were used to select transformed *E. coli* BL21-Star^TM^ (DE3) transformations. Then, three bacterial colonies harboring each expression plasmid were cultured overnight in Luria-Bertani medium (LB) supplemented with kanamycin (0.05 mg mL^−1^) on a rotating shaker at 37 °C until they reached an optical density (OD) of 0.4 at 600 nm. Isopropyl-ß-D-1-thiogalactopyranoside (IPTG, a final concentration of 0.4 mM; Sigma, St. Louis, MO, USA) was then added to the culture, and the induced cultures were allowed to grow for 4 h. Cells were ruptured on ice using ultrasonic sonication, and debris was eliminated through centrifugation (20,000× *g* for 30 min at 4 °C). Under denaturing conditions and per the manufacturer’s instructions, the recombinant proteins were purified using Ni Sepharose High Performance immobilized metal ion affinity chromatography columns (HisTrap Hp, cat. n. GE17-5248-02, GE Healthcare, Logan, UT, USA) coupled to an ÄKTA Pure (GE Healthcare, Chicago, IL, USA).

### 2.2. GM-CSF-Based DNA Vaccine

The GM-CSF amino acid sequence was sent to FastBio (Ribeiro Preto, Brazil) for eukaryotic cell optimization and gene synthesis. The synthetic genes were cloned in the pUC57 vector before being sub-cloned into the pCI-neo mammalian expression vector insert (Promega Incorporation, Madison, WI, USA). *Escherichia coli* DH5α was transformed using pCI-GM-CSF, and the immunization plasmid was purified according to the manufacturer’s instructions using the ZR plasmid Gigaprep Kit (Zymo Research, Irvine, CA, USA), as previously described [24].

### 2.3. Liposome Preparation and Entrapment of Plasmid DNA

In the present study, we employed a cationic liposome to deliver the GM-CSF DNA plasmid DNA effectively, as previously described by Fotoran [28] with minor modifications [24]. Briefly, dimethyl-di-octadecyl-ammonium (DDAB), 1,2-distearoyl-sn-glycero-3-phosphoethanolamine-N-[amino (polyethylene glycol)-2000 (DSPE + PEG2000), cholesterol (molar ratio 1:4), 5% of the total lipids were used to produce liposomes. This lipid solution was dissolved in 1 mL of chloroform before being exposed to a steady flow of N_2_ to evaporate the chloroform and generate a phospholipid layer on the tube walls. To remove all traces of chloroform, this film was then kept under vacuum for at least an hour. Afterward, the film was rehydrated in 5 mM Tris-HCl (pH 7.5) at 60 °C for 1 h while being stirred vigorously every 10 min. After obtaining an opaque solution, we subjected it to high-energy sonication until it approached complete translucence, and then the solution was centrifuged at 100,000× *g* for 1 h. Any remaining pellet was eliminated, and the supernatant containing unilamellar lipid vesicles was employed to produce the liposome. The genetic material was subsequently added to the liposomes using a molar stoichiometry of 8 nM DDAB per 1 g of nucleic acids, with an estimated molarity of 0.2 pmol.

### 2.4. S. aureus Antigens Candidates: Immunological Evaluation in Mice

**Figure 1:**
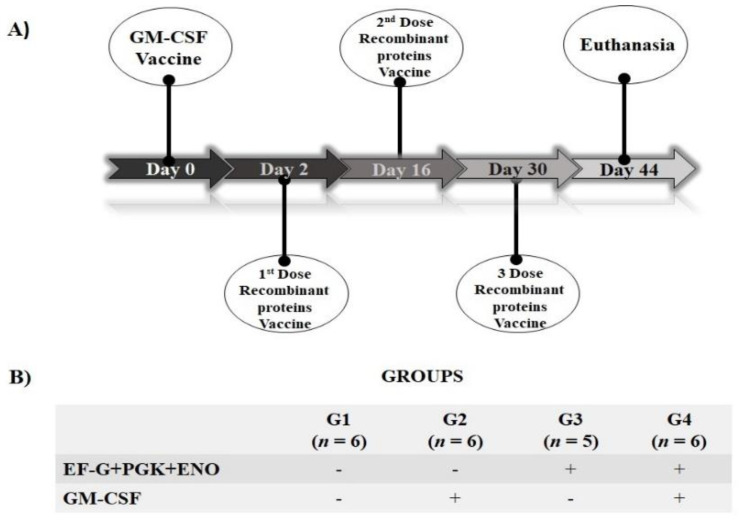
Scheme of the timeline of immunization (**A**) and experimental groups (**B**). EF-G: elongation factor g recombinant *S. aureus* protein; PGK: transmembrane protein phosphoglycerate kinase recombinant *S. aureus* protein; ENO: enolase recombinant *S. aureus* protein; GM-CSF: pCI-granulocyte and macrophage colony-stimulating factor plasmid DNA. The unvaccinated group was merely administrated saponin adjuvant to liposome.

In the present study, twenty-three female, six-week-old C57BL/6J mice purchased from the Centro de Bioterismo, of Faculdade de Medicina, Universidade de São Paulo, were divided, after three weeks of adaptation, into four groups and immunized as described in Figure 1. In the present study, 50 µg of pCI-GM-CSF plasmid DNA resuspended in 100 µL of sterile liposomal formulation were injected subcutaneously near the draining lymph nodes in the interscapular region. The recombinant EF-G, PGK, and ENO proteins (20 g each) were injected intramuscularly into the deltoid muscles in 100 µL of saponin adjuvant (Quil-A^®^, cat. n. 8047-15-2, Invivogen, San Diego, CA, USA) (Figure 1).

On day 44 (14 days following the last dose of *S. aureus* recombinant proteins), the mice were anesthetized intramuscularly with a solution of xylazine (10 mg kg^−1^) and ketamine (50 mg kg^−1^) and euthanized by cervical dislocation.

### 2.5. Spleen and Cell Recovery

The spleen was then excised to obtain splenic cells for further immunophenotyping and lymphocyte proliferation studies, as previously described [24]. The spleens were mechanically disrupted and homogenized with a syringe plunger into a cell strainer (cat. n. Z742102, Sigma Aldrich, St. Louis, MO, USA) on top of a 50 mL tube containing 10 mL of RPMI 1640 medium (cat. n. R7638, Sigma Aldrich, St. Louis, MO, USA) supplemented with 5% heat-inactivated fetal bovine serum (Gibco, Waltham, MA, USA), 100 μg mL^−1^ streptomycin, 100 U mL^−1^ penicillin and Fungizone 0.25 μg mL^−1^ (cat. n. 15240-096, Gibco, Waltham, MA, USA) (complete medium).

The spleen and lymph node cells were centrifuged for 10 min at 4 °C at 380× *g*. Erythrocytes were lysed hypotonically by introducing 1000 μL of 0.2% NaCl for 20 s, and afterwards, isotonicity was reestablished by adding 1000 μL of 1.6% NaCl. The cells were centrifuged for 10 min at 4 °C at 380× *g* and then resuspended in 1 mL of the complete medium. The viability of cells was initially determined using the trypan blue exclusion test (cat. n. T8154-100ML, Sigma Aldrich, St. Louis, MO, USA).

### 2.6. Preparation of S. aureus Inoculum

*Staphylococcus aureus* (spa typing t605) isolated from a case of persistent subclinical IMI was used, and the bacterial inoculum dose (2 × 10^8^ staphylococci mL^−1^) was prepared as previously described [29] with minor modifications [24]. Briefly, bacteria were resuspended in a complete medium after measuring the colony-forming unit, adjusting the concentration of the bacterial inoculum to achieve a ratio of 10 bacteria per cell, and inactivating the bacteria for 1 h at 60 °C.

### 2.7. Immunophenotyping and Lymphocyte Proliferation Evaluation of Cultured Splenic Cells

The lymphocyte proliferation of splenic cells with or without (control) 10 µL of heat-inactivated *S. aureus* (2 × 10^8^ CFU mL^−1^; 10 bacteria per cell) was measured using 2 × 10^5^ viable cells per well (96-wells flat-bottom plates) at 37 °C and 5% CO_2_ atmosphere for 96 h. The immunophenotyping and lymphocyte proliferation were performed as previously described [24]. The supernatant of the cell culture was collected and frozen at −80 ℃ for cytokines quantification. Briefly, the splenic cells were immunophenotyped using fluorescent-conjugated monoclonal antibodies (mAbs; Table 1). Then, the cells were fixed and permeabilized for assessment of intracellular production of interferon (IFN)-γ and interleukin (IL)-17A, besides the evaluation of in vitro antigen-specific lymphocyte proliferation using ki67 [24,30,31]. The samples were analyzed by flow cytometry (BD LSRFortessa^TM^ X-20 flow cytometer, Becton Dickinson Immunocytometry System^TM^, San Diego, CA, USA). Here, 100,000 events were assessed per sample. The data were analyzed using Flow Jo Tree Star software (FlowJo–Treestar 10.5.3 for Windows, Tree Star Inc., Ashland, OR, USA). As compensation controls, an unstained control, and single-stained samples were also prepared. Conjugated isotype control antibodies were also used in negative control samples. Furthermore, splenic cells were stained with fluorescence minus-one (FMO) controls. The forward scatter (FSC) area versus FSC height was used to exclude doublets.

### 2.8. Cytokine Measurement

The production rates of cytokines IFN-γ, tumor necrosis factor (TNF)-α, IL-17A, IL-2, IL-4, IL-6 and IL-10 were measured in the supernatant of control or *S. aureus*-stimulated spleen cell culture by the BD Cytometric Bead Array Mouse T_H_1, T_H_2 and T_H_17 Cytokine (cat. n. 560485, BD Bioscience^TM^, San Jose, CA, USA) using a flow cytometer (BD LSR Fortessa^TM^ X-20 flow cytometer, Becton Dickinson Immunocytometry System^TM^, San Diego, CA, USA) following the manufacturer’s instructions. The data were analyzed using the FCAP Array^TM^ v3.0 software (Softflow^TM^, Pécs, Hungary).

### 2.9. Statistical Analysis

To accurately measure the intensity of T cell response upon *S. aureus* stimulation, the data were normalized by dividing the percentage of proliferative cells (ki67^+^) or the geometric mean fluorescence (GMFI) upon *S. aureus* stimulation by the percentage of proliferative cells (ki67^+^) or the GMFI of unstimulated control, thereby generating a stimulation index (SI) [24,32,33,34,35,36]. The GMFI values of IL-17A or IFN-γ were measured among IL-17A^+^ or IFN-γ^+^ cells to determine the intensity of cytokine production per cell. The Gaussian distribution was verified using the Kolmogorov–Smirnov and Shapiro–Wilk tests. A one-way ANOVA was performed on the parametric data, followed by the Tukey test. The Kruskal–Wallis test, followed by Dunn’s test, was used to assess variables with non-parametric distributions. Results are reported as violin plots (median, interquartile range, and adjacent values). *p* ≤ 0.05 was set as significant unless otherwise indicated. GraphPad Prism 9 (GraphPad Software, Inc., San Diego, CA, USA) was used to perform the statistical analysis.

## 3. Results

### 3.1. Type 3 Immunity Is Triggered by αβ and γδ T Memory Cells in Immunized Animals with the Three Recombinant Proteins

**Figure 2:**
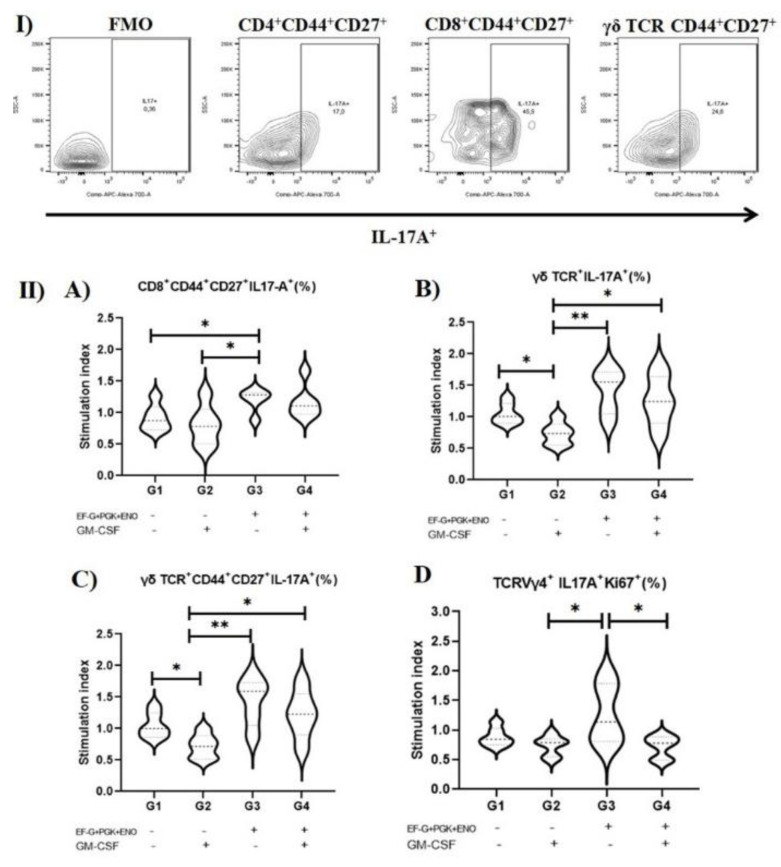
Percentage of IL-17A^+^ T cells in mice vaccinated with three *S. aureus*-cure-associated recombinant proteins. T cell responses are normalized by dividing the percentage of IL-17A^+^ cells upon *S. aureus* stimulation per the percentage of IL-17A^+^ cells under unstimulated control a stimulation index. (**I**) Representative gating of IL-17A production by different lymphocyte populations. (**II**) Stimulation index of (**A**) T CD8^+^ CD44^+^ CD27^+^ (central memory) cells, (**B**) overall γδ TCR^+^ cells, (**C**) γδ TCR^+^ CD44^+^ CD27^+^ (central memory) cells, and (**D**) TCRVγ4^+^ Ki67^+^ cells. Granulocyte-macrophage colony-stimulating factor; EF-G: elongation factor-G, ENO: enolase; and PGK: phosphoglycerate kinase. * indicate *p* ≤ 0.05, ** indicate *p* ≤ 0.01. (One-way ANOVA followed by Tukey test).

Immunization with the three *S. aureus* recombinant proteins alone resulted in a higher SI of the IL-17A^+^ cells among CD8^+^ T_CM_ cells (*p* = 0.04) compared with the non-immunized control group and with the GM-CSF DNA vaccine group (Figure 2). Further, animals immunized with the three *S. aureus* recombinant proteins alone had a higher SI of IL-17A^+^ cells among proliferative (Ki67^+^) TCRVγ4^+^ compared with those that received the GM-CSF DNA vaccine (Figure 2). In contrast, GM-CSF DNA vaccination alone negatively impacted the SI of IL-17A^+^ cells among γδ T cells (*p* = 0.005) and γδ central memory T cells (T_CM_) (*p* = 0.005) compared with non-immunized control (Figure 2). No effect of immunization on type 1 (IFN-γ^+^) immune cells was observed.

**Figure 3:**
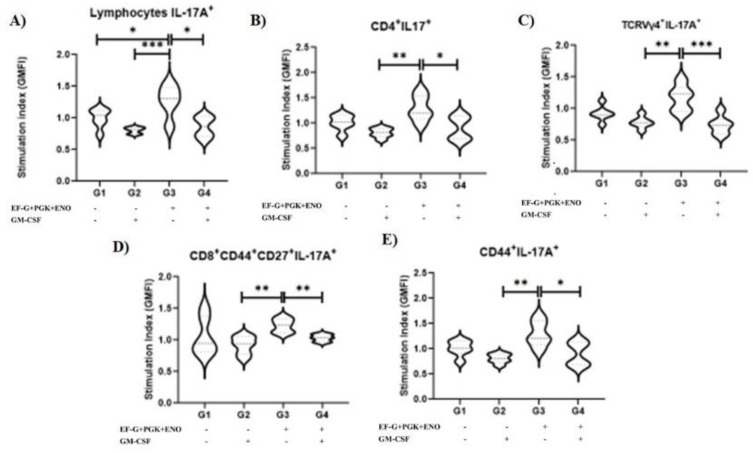
Geometric mean fluorescence intensity (GMFI) of IL-17A^+^ production by T cells in mice vaccinated with three-antigens *S. aureus*-cure-associated recombinant proteins. T cell responses are normalized by dividing the GMFI of IL-17A^+^ cells upon *S. aureus* stimulation per the percentage of IL-17A^+^ cells under unstimulated control condition, thereby creating a stimulation index. Here, we show the GMFI of stimulation index of (**A**) overall lymphocytes, (**B**) T CD4^+^, (**C**) TCRVγ4^+^ and (**D**) T CD8^+^ CD44^+^ CD27^+^ (central memory) and (**E**) T CD44^+^ lymphocytes. Granulocyte-macrophage colony-stimulating factor; EF-G: elongation factor-G, ENO: enolase; and PGK: phosphoglycerate kinase. * indicate *p* ≤ 0.05, ** indicate *p* ≤ 0.01, *** indicate *p* ≤ 0.001 (One-way ANOVA followed by Tukey test).

Importantly, we observed the highest SI values in the intensity of IL-17A production by overall lymphocytes in animals immunized with the combination of recombinant proteins compared with other groups (*p* = 0.05; Figure 3). These data suggest that the contribution of the combined lymphocyte populations is crucial to sustaining a type 3 cell immunity environment. However, analyzing distinct populations, we only detected a tendency to higher SI in the intensity of IL-17A production in animals immunized with the combination of recombinant proteins in T CD4^+^ lymphocytes, TCRVγ4^+^ cells, T CD8^+^ central memory cells and in overall memory T cells when compared with both groups that received the GM-CSF DNA vaccine (Figure 3).

**Figure 4:**
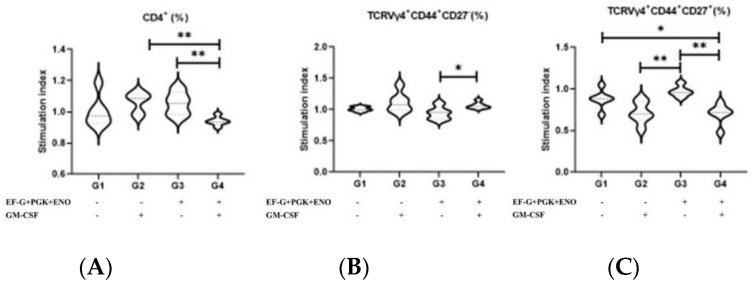
Percentage of T CD4^+^ cells in immunized animals, and the antidromic trend of the percentage TCRVγ4^+^ effector and central memory cells in mice vaccinated with GM-CSF DNA vaccine. (**A**) Overall T CD4^+^, (**B**) TCRVγ4^+^ CD44^+^ CD27^−^ (effector memory cells), and (**C**) TCRVγ4^+^ CD44^+^ CD27^+^ (central memory cells). GM-CSF: Granulocyte-macrophage colony-stimulating factor; EF-G: elongation factor-G, ENO: enolase; and PGK: phosphoglycerate kinase. * Indicate *p* ≤ 0.05, ** indicate *p* ≤ 0.01. (One-way ANOVA followed by Tukey test).

The animals that received the combination of three *S. aureus* recombinant proteins and the GM-CSF DNA vaccine had a lower SI of T CD4^+^ cells than the other vaccinated groups (*p* = 0.03), although it did not significantly differ from the unvaccinated control animals (Figure 4). The GM-CSF DNA vaccination favors an increase in the SI of TCRVγ4^+^ effector memory cells (*p* = 0.06) while dampening TCRVγ4^+^ central memory cells (*p* = 0.001; Figure 4). No B cell response was observed upon immunization (data not shown).

### 3.2. Animals Immunized with Three Recombinant Proteins in Association with the GM-CSF DNA Vaccine Was Associated an Anti-Inflammatory Cytokines Production

No effect of immunization on the levels of IL-2 and IL-4 cytokines was observed. The levels of IL-6 (*p* = 0.06) tend to be lower upon stimulation with *S. aureus* than unstimulated control just in immunized animals with the three recombinant proteins and the GM-CSF DNA vaccine (Supplementary Figure S1); however, no significant difference among groups was observed under unstimulated control (*p* = 0.79) and *S. aureus* (*p* = 0.68) stimulation conditions. The concentrations of IL-10, IL-17A, TNF-α and IFN-γ were below the limit of the detection.

## 4. Discussion

We should highlight that our previous study has already shown that specific antibodies (humoral immunity) against the three *S. aureus* proteins used here are related to the cure of existing IMIs by *S. aureus* after vaccination with a commercial (bacterin) vaccine [7]. However, humoral immunity alone may not provide adequate protection against *S. aureus* infections [26]. In this context, there is an emerging knowledge appointing that memory T cells critically contribute to the *S. aureus* infection control [37]. Indeed, deficiencies in T cells enhance susceptibility to *S. aureus* infections [37,38]. Interestingly, protective immunity against recurrent *S. aureus* skin and soft tissue infection in a human was associated with both antibody and T-cell mediated (i.e., IL-17) immunities, suggesting that a multimechanistic approach targeting both humoral and T-cell-mediated immunities may be an important strategy for providing protective immunity and preventing new *S. aureus* infections [26]. Furthermore, Interleukin-17 is associated with antigen-specific inflammation, neutrophil recruitment, and modulating the communication between the immune system and mammary epithelial cells [39]. Puzzling, the investigation on T-cell-mediated immunity, especially on type 3 immunity, in *S. aureus* vaccine development against bovine mastitis had not received much attention so far. In the present study, the data suggest that both αβ and γδ T cells contribute to type 3 immunity, which has been regarded as the major mechanism of mammary gland immune defense [39]. Type 3 immunity is mediated by a variety of cells, including γδ T cells, T CD4^+^ helper (Th17), T CD8^+^ (Tc17) and innate lymphoid cells 3 [39]. A population of highly IL-17A-producing memory TCRVγ4^+^ cells has recently been recognized as the most important population that confers protective immunity against subsequent infections by *S. aureus* [40,41]. In the present study, we observed a tendency for higher SI in this subpopulation. Therefore, we assumed that overall IL-17A highly producing αβ and γδ T cells are required for spontaneous recovery from IMI caused by *S. aureus*, rather than a specific γδ T cell subpopulation, as we found here. Thus, our study focusing on three targets of *S. aureus* recombinant protein immunization places the basis for the increases in the likelihood of the cure of an existing *S. aureus* IMI in an integrated approach with antimicrobial treatment or a non-antibiotic approach to deal with existing *S. aureus* IMIs, especially regarding the refractoriness of this pathogen to the current antimicrobial therapies, although further studies in dairy cows are still needed.

Although GM-CSF is an important mediator of the immune response [42], our outcomes did not show robust improvement in T-cell mediated immunity of the three *S. aureus-*cure-associated antigens boosted by the GM-CSF DNA vaccine. In this concern, animals that were vaccinated with the GM-CSF DNA vaccine had a lower SI of IL-17A^+^ cells among overall memory (CD44^+^) proliferative lymphocytes and TCRVγ4^+^ proliferative cells, which could have a critical implication for vaccine efficacy.

Although we do not have any clear idea of this paradox, it has been postulated that it depends on numerous factors, including the immunogen(s), the dose and the timing of GM-CSF administration [43]. In this regard, it has been proposed that great amounts of GM-CSF may expand myeloid cells in secondary lymphoid organs, which in turn may recruit T regulatory cells leading to an immunosuppressive effect [43]. One factor that may result in this effect is the use of a cationic liposome-based approach to efficiently deliver the GM-CSF DNA plasmid DNA.

## 5. Conclusions

The immunization with three *S. aureus-*cure-associated recombinant proteins triggered the type 3 immune response in splenocytes stimulated with *S. aureus* by overall T cells, which is a highly interesting path to pursue an effective control strategy against bovine *S. aureus* mastitis. Therefore, although our results are encouraging, further studies are required to corroborate our findings about the protective immunity against *S. aureus* in ruminants triggered by these recombinant *S. aureus* proteins.

## 6. Patent

The authors have a patent titled “Vaccine composition, kit for diagnosis of *Staphylococcus aureus* infections in ruminants, method and use.”

## Figures and Tables

**Table 1:** Monoclonal antibodies (mAbs) used for immunophenotyping of spleen lymphocytes by flow cytometry.

mAbs	Fluorescent Probes	Clone	Host	Cat. n.
Anti-IL-17A ^1^	Alexa 700	TC11-18H10	Rat	554,412
Anti-IFN-γ ^1^	PE	XMG1.2	Rat	554,412
Anti-TCRVγ4 ^1^	FITC	GL2	Hamster	552,143
Anti- γδ TCR ^1^	BV650	GL3	Hamster	563,993
Anti-CD4 ^1^	APC-Cy7	GK1.5	Rat	552,051
Anti-CD8 ^1^	BV510	53-6.7	Rat	563,068
Anti-CD44 ^1^	BV421	IM7	Rat	563,970
Anti-CD27 ^1^	BV750	LG.3A10	Hamster	747,399
Anti-CD19 ^1^	PE-Cy7	1D3	Rat	552,854

^1^ BD Pharmingen™ (San Diego, CA, USA); IL-17A: interleukin-17A; IFN-γ: interferon-γ; FITC: Fluorescein isothiocyanate; APC-Cy7: Allophycocyanin-cyanine 7; PE: R-Phycoerythrin; PE-Cy7: R-Phycoerythrin-cyanine 7; BV510: Brilliant Violet 510; BV421: Brilliant Violet 421; BV650: Brilliant Violet 650; BV750: Brilliant Violet 750.

## Data Availability

The data presented in this study are available in the manuscript and the Supplemental File. Additional data are available upon reasonable request from the corresponding author.

## Supplementary Materials

The following supporting information can be downloaded at: https://www.mdpi.com/article/10.3390/antibiotics11121831/s1, Figure S1: Interleukin-6 (IL-6) concentration in the supernatant of the immunized and non-immunized mouse spleen cells culture under unstimulated (basal) and *Staphylococcus aureus* stimulated conditions. G1: unvaccinated control group; G2: GM-CSF DNA plasmid DNA vaccination; G3: EF-G + ENO + PGK *S. aureus* recombinant proteins vaccination; G4: EF-G + ENO+ PGK *S. aureus* recombinant proteins vaccination associated with GM-CSF DNA plasmid DNA vaccine; EF-G: elongation factor-G; ENO: enolase; and PGK: phosphoglycerate kinase. [Student paired *t*-test].
