# Supplementary material for: Staphylococcus aureus-Cure-Associated Antigens Elicit Type 3 Immune Memory T Cells
Source: Antibiotics (Basel). 2022 Dec 16;11(12):1831. doi: 10.3390/antibiotics11121831 (PMC9774748; doi:10.3390/antibiotics11121831)
Supplement: Supplementary file 1 [file antibiotics-11-01831-s001.zip › antibiotics-1977386-supplementary.pdf]

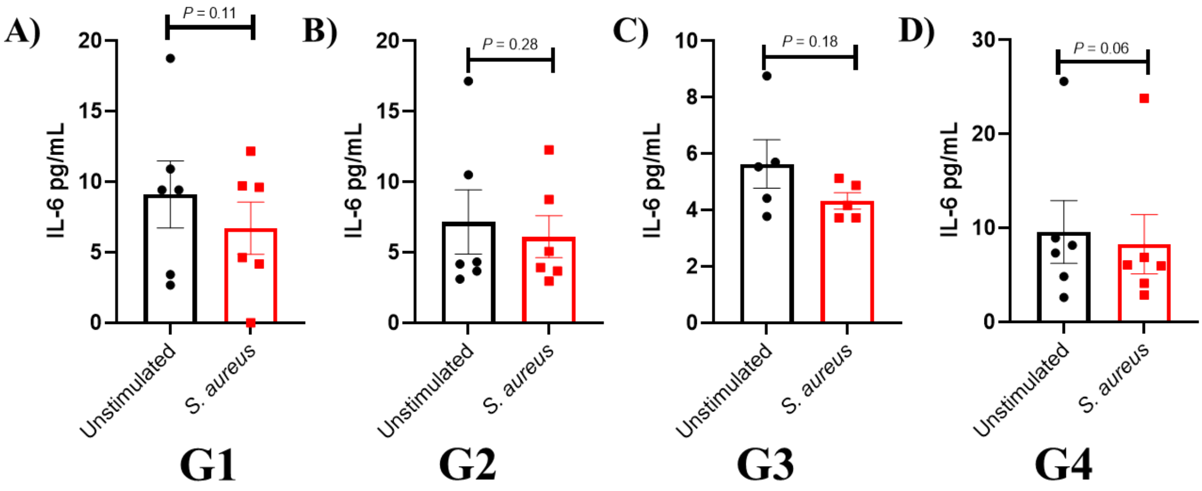

Figure S1. Interleukin-6 (IL-6) concentration in the supernatant of the immunized and non-immunized mouse spleen cells culture under unstimulated (basal) and *Staphylococcus aureus* stimulated conditions. G1: unvaccinated control group; G2: GM-CSF DNA plasmid DNA vaccination; G3: EF-G + ENO + PGK *S. aureus* recombinant proteins vaccination; G4: EF-G + ENO+ PGK *S. aureus* recombinant proteins vaccination associated with GM-CSF DNA plasmid DNA vaccine; EF-G: elongation factor-G; ENO: enolase; and PGK: phosphoglycerate kinase. [Student paired t-test].
